# Supplementary material for: Comparing animal well-being between bile duct ligation models
Source: PLoS One. 2024 Jul 1;19(7):e0303786. doi: 10.1371/journal.pone.0303786 (PMC11216573; doi:10.1371/journal.pone.0303786)
Supplement: S1 Fig — The percentage of necrotic areas (A) was assessed in healthy mice (control) after cBDL and within the ligated left liver lobes of pBDL mice (pBDL-LL). Necrosis was also evaluated (B) after ligating the left bile duct without injuring the left hepatic artery (called verified partial bile duct ligation, v-pBDL) or after ligating the left bile duct plus the left hepatic artery (pBDL+pAL-LL). Kruskal Wallis test (ANOVA on ranks) with Dunn’s correction (A) and Mann-Whitney test (B). *P < 0.05 is considered to be statistically significant. The median + 95% CI is shown; control: n = 6, cBDL: n = 9, pBDL-LL: n = 14. (DOCX) [file pone.0303786.s001.docx]

**
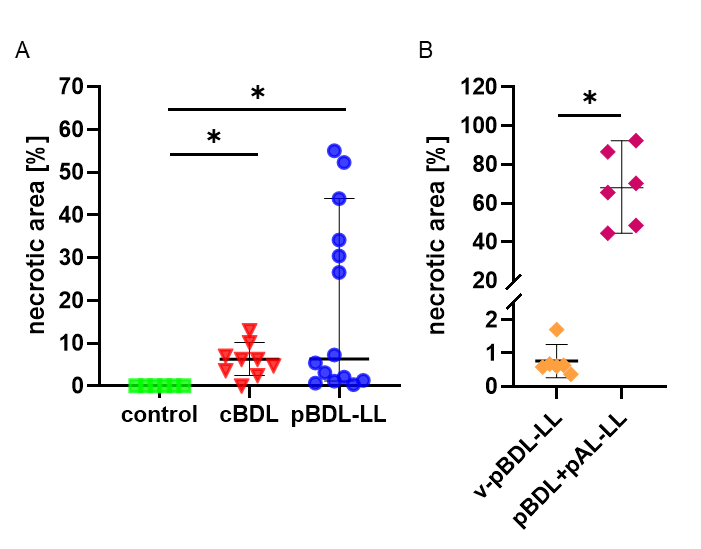
**

**S1 Fig. Quantification of liver damage.** The percentage of necrotic areas (A) was assessed in healthy mice (control) after cBDL and within the ligated left liver lobes of pBDL mice (pBDL-LL). Necrosis was also evaluated (B) after ligating the left bile duct without injuring the left hepatic artery (called verified partial bile duct ligation, v-pBDL) or after ligating the left bile duct plus the left hepatic artery (pBDL+pAL-LL). Kruskal Wallis test (ANOVA on ranks) with Dunn’s correction (A) and Mann-Whitney test (B). *P < 0.05 is considered to be statistically significant. The median + 95 % CI is shown; control: n = 6, cBDL: n = 9, pBDL-LL: n = 14.
