# Supplementary material for: Comparing animal well-being between bile duct ligation models
Source: PLoS One. 2024 Jul 1;19(7):e0303786. doi: 10.1371/journal.pone.0303786 (PMC11216573; doi:10.1371/journal.pone.0303786)
Supplement: S2 Fig — After cBDL or v-pBDL the survival rate (A), the distress score (B), body weight (C), and burrowing activity (D) were evaluated during the pre-operative phase (pre) as well as during early (e), middle (m) and late (l) phase of cholestasis. In B, C and D the significance was determined by a Two-Way RM ANOVA. Sidak Test for multiple comparisons when comparing between the two ligation methods at each time point (*P < 0.05). Dunnett test for multiple comparisons when compared to pre-experimental time point within the cBDL group (#P < 0.05) and within the v-pBDL group (##P < 0.05). The median + 95% CI is shown; cBDL: n = 9, v-pBDL: n = 6 animals. (DOCX) [file pone.0303786.s002.docx]

**
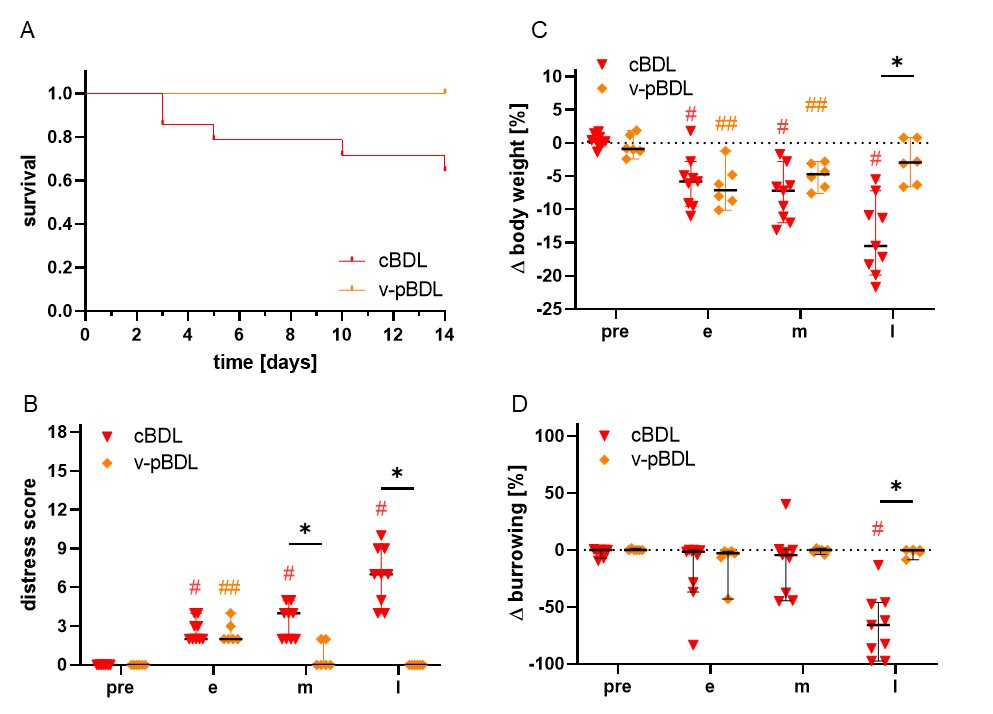
**

**S2 Fig. Survival and distress of mice after cBDL or v-pBDL.** After cBDL or v-pBDL the survival rate (A), the distress score (B), body weight (C), and burrowing activity (D) were evaluated during the pre-operative phase (pre) as well as during early (e), middle (m) and late (l) phase of cholestasis. In B, C and D the significance was determined by a Two-Way RM ANOVA. Sidak Test for multiple comparisons when comparing between the two ligation methods at each time point (*P < 0.05). Dunnett test for multiple comparisons when compared to pre-experimental time point within the cBDL group (^#^P < 0.05) and within the v-pBDL group (^##^P < 0.05). The median + 95 % CI is shown; cBDL: n = 9, v-pBDL: n = 6 animals.
