# Supplementary material for: Comparing animal well-being between bile duct ligation models
Source: PLoS One. 2024 Jul 1;19(7):e0303786. doi: 10.1371/journal.pone.0303786 (PMC11216573; doi:10.1371/journal.pone.0303786)
Supplement: S3 Fig — Relative expression (2(-ΔΔCT)) of TNFα (A) and IFNγ (B) in the liver of healthy mice (control), after cBDL and within the ligated left liver lobe of v-pBDL mice (v-pBDL-LL). Kruskal Wallis test (ANOVA on ranks) with Dunn’s correction for multiple comparisons (*P < 0.05). The median + 95% CI is shown; control: n = 6, cBDL: n = 9, v-pBDL: n = 6 animals. (DOCX) [file pone.0303786.s003.docx]

**
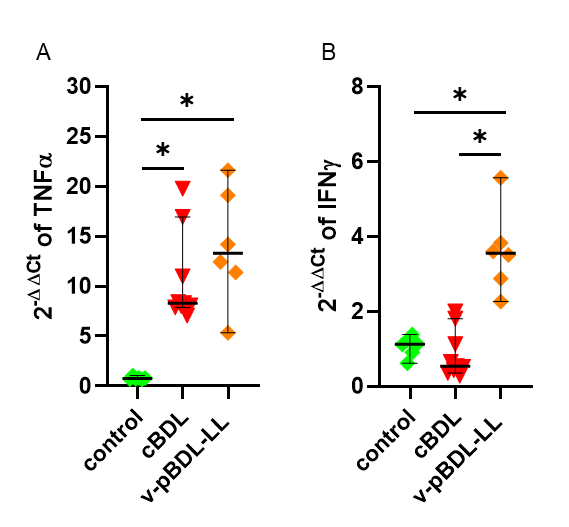
**

**S3 Fig. Cytokine expression in cBDL and v-pBDL mice.** Relative expression (2^(-ΔΔCT)^) of *TNFα* (A) and *IFNγ* (B) in the liver of healthy mice (control), after cBDL and within the ligated left liver lobe of v-pBDL mice (v-pBDL-LL). Kruskal Wallis test (ANOVA on ranks) with Dunn’s correction for multiple comparisons (*P < 0.05). The median + 95 % CI is shown; control: n = 6, cBDL: n = 9, v-pBDL: n = 6 animals.
