# Supplementary material for: Comparing animal well-being between bile duct ligation models
Source: PLoS One. 2024 Jul 1;19(7):e0303786. doi: 10.1371/journal.pone.0303786 (PMC11216573; doi:10.1371/journal.pone.0303786)
Supplement: S4 Fig — Direct (A) and indirect bilirubin (B) in blood plasma of healthy mice (control), after cBDL and v-pBDL. Since in many samples bilirubin was below the detection limit, no statistical evaluation was done. The median + 95% CI is shown; control: n = 12, cBDL: n = 9, v-pBDL: n = 6 animals. (DOCX) [file pone.0303786.s004.docx]

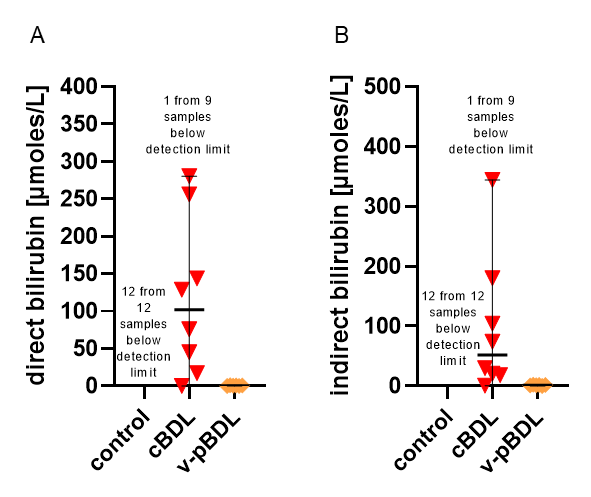


**S4 Fig. Direct and indirect bilirubin.** Direct (A) and indirect bilirubin (B) in blood plasma of healthy mice (control), after cBDL and v-pBDL. Since in many samples bilirubin was below the detection limit, no statistical evaluation was done. The median + 95 % CI is shown; control: n = 12, cBDL: n = 9, v-pBDL: n = 6 animals.
