# Supplementary material for: Comparing animal well-being between bile duct ligation models
Source: PLoS One. 2024 Jul 1;19(7):e0303786. doi: 10.1371/journal.pone.0303786 (PMC11216573; doi:10.1371/journal.pone.0303786)
Supplement: S5 Fig — Macroscopic images of liver show necrosis (black arrows) on day 14 after v-pBDL (A) or pBDL+pAL (B). (DOCX) [file pone.0303786.s005.docx]

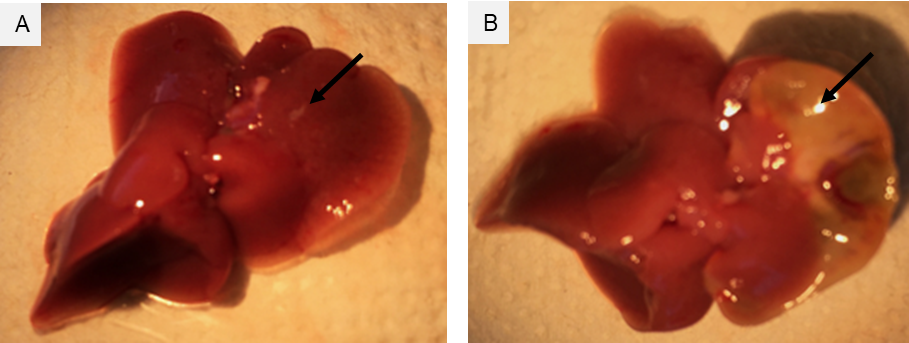


**S5 Fig. Liver necrosis.** Macroscopic images of liver show necrosis (black arrows) on day 14 after v-pBDL (A) or pBDL+pAL (B).
