# Supplementary material for: Comparing animal well-being between bile duct ligation models
Source: PLoS One. 2024 Jul 1;19(7):e0303786. doi: 10.1371/journal.pone.0303786 (PMC11216573; doi:10.1371/journal.pone.0303786)
Supplement: S6 Fig — Histological sections of the median liver lobes of v-pBDL (v-pBDL-ML) and pBDL+pAL (pBDL+pAL-ML) mice were analyzed. Hämatoxylin/eosin stained sections (A, B), the percentage of necrotic area (C), representative images after collagen I immunohistochemistry (D, E), the percentage of collagen I+ area (F), chloroacetate esterase stained sections (G, H) and its quantification (I) are presented. No significant differences were observed using Kruskal Wallis test (ANOVA on ranks) with Dunn’s correction. The median + 95% CI is shown; control: n = 6, v-pBDL-ML: n = 6, pBDL+pAL-ML: n = 6 animals. (DOCX) [file pone.0303786.s006.docx]

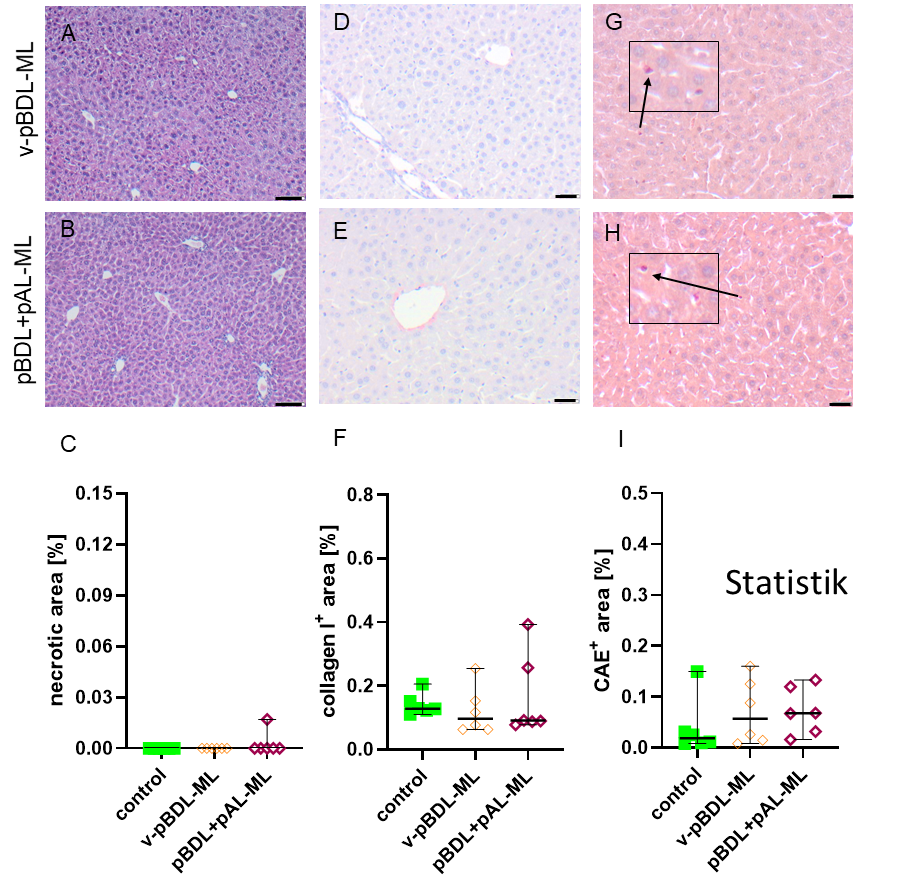


**S6 Fig. Evaluation of necrosis, fibrosis, and inflammation in unligated liver lobes.** Histological sections of the median liver lobes of v-pBDL (v-pBDL-ML) and pBDL+pAL (pBDL+pAL-ML) mice were analyzed. Hämatoxylin/eosin stained sections (A, B), the percentage of necrotic area (C), representative images after collagen I immunohistochemistry (D, E), the percentage of collagen I^+^ area (F), chloroacetate esterase stained sections (G, H) and its quantification (I) are presented. No significant differences were observed using Kruskal Wallis test (ANOVA on ranks) with Dunn’s correction. The median + 95 % CI is shown; control: n = 6, v-pBDL-ML: n = 6, pBDL+pAL-ML: n = 6 animals.
