# Supplementary material for: Comparing animal well-being between bile duct ligation models
Source: PLoS One. 2024 Jul 1;19(7):e0303786. doi: 10.1371/journal.pone.0303786 (PMC11216573; doi:10.1371/journal.pone.0303786)
Supplement: S8 Fig — Relative expression (2(-ΔΔCT)) of TNFα (A) and IFNγ (B) in the liver of healthy mice (control), and in the unligated medium liver lobes of v-pBDL (v-pBDL-ML) and pBDL+pAL (pBDL+pAL-ML) mice. No significant differences according to Kruskal Wallis test (ANOVA on ranks) with Dunn’s correction (A) or one-way ANOVA (B) with Tukey correction for multiple comparisons. The median + 95% CI is shown; control: n = 6, v-pBDL: n = 6, pBDL+pAL: n = 6 animals. (DOCX) [file pone.0303786.s008.docx]

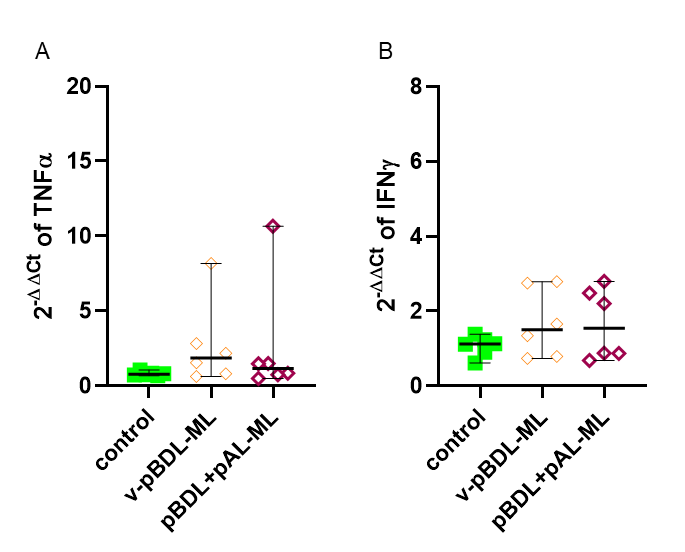


**S8 Fig. Expression of cytokines in unligated liver lobes.** Relative expression (2^(-ΔΔCT)^) of TNFα (A) and IFNγ (B) in the liver of healthy mice (control), and in the unligated medium liver lobes of v-pBDL (v-pBDL-ML) and pBDL+pAL (pBDL+pAL-ML) mice. No significant differences according to Kruskal Wallis test (ANOVA on ranks) with Dunn’s correction (A) or one-way ANOVA (B) with Tukey correction for multiple comparisons. The median + 95 % CI is shown; control: n = 6, v-pBDL: n = 6, pBDL+pAL: n = 6 animals.
