# Supplementary material for: Comparing animal well-being between bile duct ligation models
Source: PLoS One. 2024 Jul 1;19(7):e0303786. doi: 10.1371/journal.pone.0303786 (PMC11216573; doi:10.1371/journal.pone.0303786)
Supplement: S9 Fig — Direct (A) and indirect bilirubin (B) in the blood plasma of mice, 14 days after v-pBDL or pBDL+pAL. No significant differences according to unpaired t-test. The median + 95% CI is shown; v-pBDL: n = 6, pBDL+pAL: n = 6 animals. (DOCX) [file pone.0303786.s009.docx]

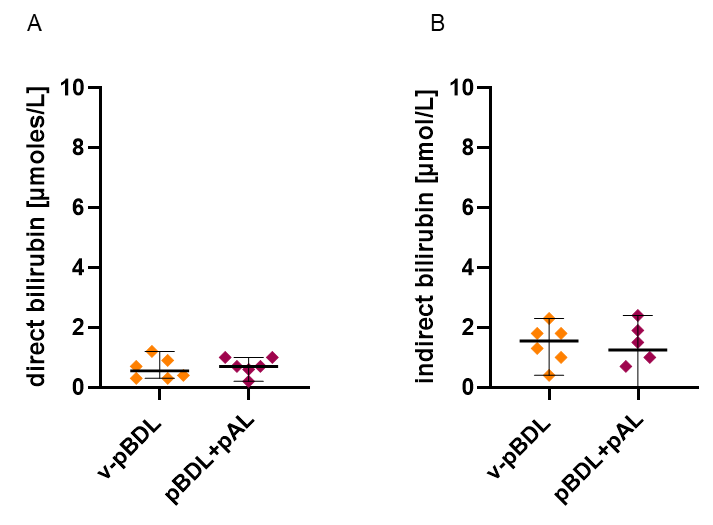


**S9 Fig. Direct and indirect bilirubin.** Direct (A) and indirect bilirubin (B) in the blood plasma of mice, 14 days after v-pBDL or pBDL+pAL. No significant differences according to unpaired t-test. The median + 95 % CI is shown; v-pBDL: n = 6, pBDL+pAL: n = 6 animals.
