# Supplementary material for: Comparing animal well-being between bile duct ligation models
Source: PLoS One. 2024 Jul 1;19(7):e0303786. doi: 10.1371/journal.pone.0303786 (PMC11216573; doi:10.1371/journal.pone.0303786)
Supplement: S10 Fig — Identification of the common bile duct (black arrow in A). For cBDL: The common bile duct (black arrow) is separated from the proper hepatic artery and the portal vein (B) and 3-fold ligated and intersected (C). For v-pBDL: ligation of the left hepatic bile duct (black arrow) without damaging accompanying artery and vein (D). (DOCX) [file pone.0303786.s010.docx]

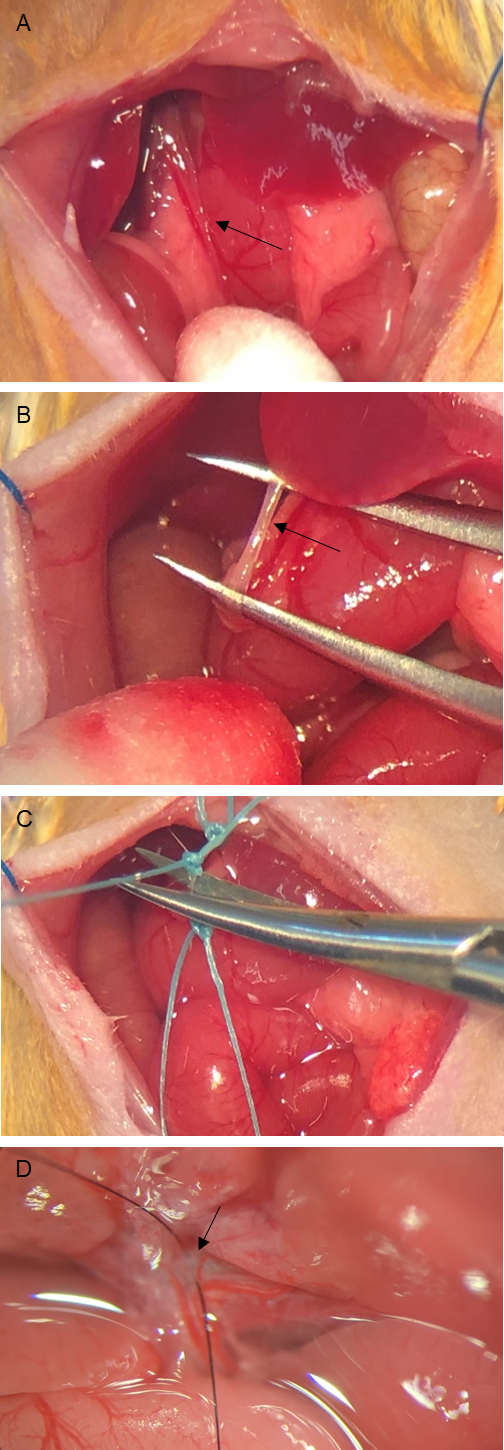


**S10 Fig. Images from distinct stages during surgical interventions.** Identification of the common bile duct (black arrow in A). For cBDL: The common bile duct (black arrow) is separated from the proper hepatic artery and the portal vein (B) and 3-fold ligated and intersected (C). For v-pBDL: ligation of the left hepatic bile duct (black arrow) without damaging accompanying artery and vein (D).
