# Supplementary material for: Comparing animal well-being between bile duct ligation models
Source: PLoS One. 2024 Jul 1;19(7):e0303786. doi: 10.1371/journal.pone.0303786 (PMC11216573; doi:10.1371/journal.pone.0303786)
Supplement: S11 Fig — At the beginning of the experiment, the mice were allowed to practice burrowing two times. Afterward, body weight (bw), the distress score (ds), and burrowing activity (b) were measured on the indicated days. On day 0, the body weight of the animals was assessed and afterwards, the bile duct ligation (BDL), either cBDL, pBDL, v-pBDL or pBDL + pAL, was performed. The blood and tissue samples were collected on day 14. Data presented in this manuscript during the pre-, early, middle, or late phase of the experiment are labelled in blue print. Please note that we present percentage change in body weight and change in burrowing activity in this mansucript (the reference data, which were used for this calculation are presented in green print). Please also note that reduced food uptake in response to a certain distress level takes one day to lead to a reduction in body weight. Therefore, body weight is measured one day after we measured the distress score. (DOCX) [file pone.0303786.s011.docx]

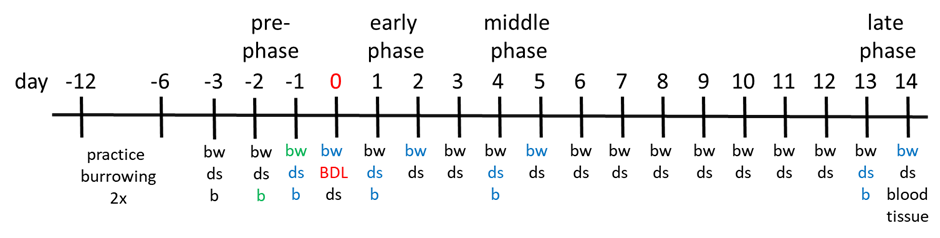


**S11 Fig: Experimental setup.** At the beginning of the experiment, the mice were allowed to practice burrowing two times. Afterward, body weight (bw), the distress score (ds), and burrowing activity (b) were measured on the indicated days. On day 0, the body weight of the animals was assessed and afterwards, the bile duct ligation (BDL), either cBDL, pBDL, v-pBDL or pBDL + pAL, was performed. The blood and tissue samples were collected on day 14. Data presented in this manuscript during the pre-, early, middle, or late phase of the experiment are labelled in blue print. Please note that we present percentage change in body weight and change in burrowing activity in this mansucript (the reference data, which were used for this calculation are presented in green print). Please also note that reduced food uptake in response to a certain distress level takes one day to lead to a reduction in body weight. Therefore, body weight is measured one day after we measured the distress score.
